# Continuing Education Needs of Occupational Health Nurses in the Mining Industry: Implications for Public Health Nursing Workforce Development

**DOI:** 10.1111/phn.70128

**Published:** 2026-04-24

**Authors:** Juli Dwi Prasetyono, Henny Permatasari, Agus Setiawan, Sigit Mulyono, Tantut Susanto, Muchtaruddin Mansyur

**Affiliations:** ^1^ Department of Nursing Science, Faculty of Nursing Universitas Indonesia Depok Indonesia; ^2^ Department of Nursing Science, Faculty of Health Sciences University of Eastern Finland Kuopio Finland; ^3^ Department of Community, Family & Geriatric Nursing, Faculty of Nursing Universitas Jember Jember Indonesia; ^4^ Department of Community Medicine, Faculty of Medicine Universitas Indonesia Depok Indonesia

**Keywords:** continuing education, focus group study, mining industry, occupational health nurse, practice

## Abstract

**Objectives:**

This study aims to (1) explore the job duties of nurses working in the mining sector, (2) identify the competencies they require to perform their roles effectively, and (3) examine organizational factors that influence their ability to provide occupational and public health services in the Indonesian mining industry.

**Design and Methods:**

An exploratory descriptive qualitative study was conducted using semi‐structured focus group interviews to investigate the participants’ experiences and expectations regarding their roles and educational needs within the mining industry.

**Sample:**

Occupational health nurses (OHNs) (*n* = 20) working in Indonesian mining sector representing different professional categories participated in semi‑structured focus group discussions (FGDs).

**Results:**

The analysis produced three overarching categories: (1) diverse public health roles and responsibilities in mining by OHNs, (2) obstacles and challenges experienced in delivering occupational health services, and (3) emerging competencies required to support workplace health and organizational productivity. Twelve themes were identified across these categories, highlighting substantial gaps between current practice demands and available educational opportunities.

**Conclusion:**

This study demonstrates that OHNs in the mining sector have expanded public health roles that often exceed their current training and routine duties. Strengthening continuing nursing education supported by the development of a formal occupational health nursing (OHN) curriculum is urgently needed to build workforce capacity. Structured education and training models, delivered through both formal and nonformal formats, should focus on communication, health data management and reporting, counseling, managerial negotiation, and interprofessional collaboration. Enhancing these competencies is vital for advancing public health nursing practice in occupational settings and improving health outcomes and productivity among mining workers.

## 1 Introduction

Public health problems in the mining industry are mainly related to noise‐induced hearing loss and respiratory diseases caused by exposure to dust and airborne contaminants (Cudjoe et al. 2025; Elkhayat et al., 2025). These issues are influenced by occupational roles, exposure duration, individual factors, such as age and smoking, and the physical characteristics of dust particles (Korshunov et al. 2025; Soltanpour et al., 2025). Moreover, miner activities impact public health through direct exposure to hazardous substances, environmental degradation, and socioeconomic changes (Cortes‐Ramirez et al., 2025; Eddy et al.2025; Lazarte et al., 2025; Shao et al. 2025). However, there is no publication on the duties of occupational health nurses (OHNs) in the mining sector. Addressing this specific need is essential for informing the development of university curricula and for strengthening continuing education (CE) opportunities delivered through both formal and nonformal training modalities.

### 1.1 Background

Beyond robust injury and fatality safety solutions, current health‐related research largely focuses on the control and suppression of various respirable dusts and aerosols that result from mining practices; work and organizational structures; design of equipment; prevention of musculoskeletal disorders (MSDs) and slips, trips, and falls; limiting noise exposure and hearing impairment; and understanding and managing the effects of both heat exposures and fatigue (CDC 2025). Miners face various health issues primarily due to the hazardous conditions associated with their poorer occupational health and safety outcomes (Jackson and Quinlan 2024). Common ailments include diseases of the ear and mastoid, vibration syndrome, MSDs, respiratory diseases, and nervous system disorders, which are prevalent among miners (Fadeev 2023). The sampled miners reported coal mining‐related pulmonary diseases, with specific conditions such as pneumoconiosis and bronchitis affecting 40% and 37%, respectively, and 66% reported cardiovascular diseases, including hypertension, hyperlipemia, and hyperglycaemia of the miners, respectively (Aram and Wang 2023). Mental health is also a significant concern for mine workers, such as anxiety, job stress, depression, and sleep disorders (Pizarro & Fuenzalida, 2021). Furthermore, the long‐term exposure to various chemical agents can lead to serious health risks, including diseases affecting the nervous and cardiovascular systems (Biyick et al. 2023).

Occupational health hazards in Indonesian mining workers include chemical exposures with potential toxic effects, heavy metal accumulation without clear disease correlation, physical work environment hazards, and a high incidence of work‐related accidents (Basri et al. 2024; Chebotarev et al.2024; Savira et al.2023; Sultan 2023; Susanto et al. 2023). A study in Malaysia reported an overall occupational respiratory disease (ORDs) prevalence of 8.7% among industrial workers, with the mining and quarrying sector showing the highest odds ratio for ORDs (AOR: 14.81) compared to other sectors (Asraff et al. 2025). In China, metal mining workers exhibited a high prevalence of occupational stress (28.8%), which was associated with adverse health outcomes, including depressive symptoms, insomnia, and work‐related musculoskeletal disorders (WMSDs) (Liu et al. 2025). A study reported the prevalence of health problems among miners in Indonesia through a comparative analysis of occupational disease diagnosis enforcement in Indonesia and other countries. In Indonesia, as in other countries, occupational diseases, such as MSDs are a significant concern (Muhammad ILYAS et al. 2025). The role of mining nurses extends beyond occupational care and directly contributes to public health nursing by managing health challenges that transcend occupational care, engaging in collaborative healthcare delivery, and addressing broader health risks within mining populations. (McCulloch & Miller, 2023; 2021, 2024; Onyeador and Umberger 2023). Currently in Indonesia, competencies in occupational health nursing (OHN) is delivered as part of the broader public health nursing curriculum rather than through a stand‐alone OHN program. Morevoer, although mining is recognized as one of the most hazardous industries worldwide, the specific contributions and evolving responsibilities of Indonesian occupational health nurse (OHNs) in this industry have not been examined in previous studies.

OHNs role in some countries ensure that miners receive appropriate health screenings, thereby enhancing their overall health outcomes (Cadet and Demezier 2025). The role of OHNs in mining environments is influenced by various factors, including the specific health risks associated with different mining operations and the implementation of health management programs (Tetzlaff et al. 2024). One key responsibility involves the identification and management of psychosocial risk factors that may affect workers' ability to recognize hazards and make informed safety decisions. Moreover, OHNs are tasked with advocating for holistic interventions that address both physical and mental health needs in the workplace (McGuire et al. 2024). Furthermore, they are involved in assessing the health impacts of mining activities on specific populations, such as adolescents in mining communities (Cambaco et al. 2025). Therefore, the study was guided by three research questions:

1. How do OHNs describe their work in the mining industry?
2. What barriers and obstacles do OHNs face in performing their job duties?
3. What expectations do OHNs have for minimizing competency gaps needed to perform their roles effectively through CE?

## 2 Design and Methods

### 2.1 Research Design

An exploratory, descriptive, and qualitative research design was undertaken in this study. A qualitative descriptive design with focus groups was used to gain a deeper understanding of the phenomenon (Creswell and Poth 2018): experiences and expectations of the OHNs to enhance health and productivity in mining companies through CNE. Focus groups are used to explore experiences because they enable interactive, 2020). participant‐led discussions that reveal detailed, context‐rich insights into personal and shared experiences, support reflexivity, and facilitate the identification of themes relevant to improving services or research engagement (2020; Collaço et al., 2025; Husabø et al.2024). The Consolidated Criteria for Reporting Qualitative Studies (COREQ) guided the designing and reporting of our study (Tong et al. 2007).

### 2.2 Sample and Setting

The data collection process was conducted at a mining holding company with 8 large mining company in Indonesia that represented the working conditions and environment in the mining industry. Unfortunately, Indonesia does not have an official national registry documenting the total number of OHNs. We relied on data from the Indonesian Occupational Health Nurse Association (INAOHN) to provide the best available estimate, which reports approximately 237 OHNs nationwide and about 112 working specifically in mining settings. In this context, our sample of 20 OHNs represents substantial coverage of the mining OHN workforce and captures variation across site types and geographic regions. In qualitative research, sample adequacy is determined by information power rather than population proportion when participants possess highly relevant experience. In this study, purposive sampling was used to recruit OHNs (*n* = 20) from total OHNs (*N* = 30) in Merdeka Company (Characteristics of the 20 OHN participants are summarised in Table 1). Purposive sampling was used to recruit OHNs with direct mining experience. Participants were identified through the Indonesian Occupational Health Nurse Association and occupational health departments in mining companies. The inclusion criteria were OHNs with two years of working experience in mining industry.

**TABLE 1:** Demographic characteristics of study participants (*n* = 20).

Characteristic	Number	Percentage (%)
**Age (years)**		
15–24	3	15
25–34	10	50
35–44	5	25
45–54	2	10
**Gender**		
Male	13	65
Female	7	5
**Length of Work (years)**		
< 5	12	60
> 5	8	40
**Education level (degree)**		
Diploma	9	45
Bachelor	11	55
Training Experience		
Not yet	2	10
Hiperkes (Occupational Health and Industrial Hygiene)	19	95
BLS (basic life support), BTLS (basic trauma and life support)	12	60
First aider	3	15

### 2.3 Data Collection

FGD is the most suitable data collection method because it is useful for exploring participants' knowledge and experience of a particular phenomenon (Kitzinger 1995). This method is suitable for exploring people's knowledge and experiences (Kitzinger 1995) regarding sensitive topics (Jordan et al. 2007), as covered in our research. Four separate semi‐structured focus group discussions (FGDs) were conducted with 4–6 OHNs in each group (total *n* = 20). Each group met once for 60–90 min. Data collection continued until saturation was reached during the fourth FGD. Researchers guide focus groups and encourage participants to participate in discussions (Nyamathi and Shuler 1990). Consent was obtained for the recording (Kitzinger 1995). Written records are kept in case of recording failure. All focus groups were conducted in Bahasa Indonesia, which is the participants’ primary and most commonly used working language. Conducting the discussions in Bahasa Indonesia enabled participants to express their experiences naturally and in detail. No interpreter was needed. Transcripts were later translated into English for reporting, and translation accuracy was checked by bilingual members of the research team to maintain meaning and ensure analytic rigor.

### 2.4 Data Analysis and Synthesis

Audio recordings are transcribed to facilitate data analysis. Data were analyzed using inductive content analysis. Inductive content analysis was chosen because of the existing knowledge of OHNs competencies are limitless (Elo and Kyngäs 2008). This method provides direct information from the study participants without using pre‐formed strategies and thus provides a richer description of the phenomenon without missing previously unidentified aspects (Hsieh and Shannon 2005). All transcripts were read repeatedly to achieve familiarization, and initial codes were generated line‑by‑line. Two researchers independently coded the first several transcripts, then compared and refined their coding to develop a shared code structure. The remaining transcripts were coded using this agreed framework in ATLAS.ti version 24, which facilitated systematic organization of codes and retrieval of data segments. Codes were then grouped into categories and abstracted into broader themes through iterative discussion within the research team. Any discrepancies were resolved through consensus, and analytic memos were maintained to document decisions and enhance rigor.

### 2.5 Ethical Considerations

Ethical approval This study received permission and recommendation from the Ethics Commission of the Faculty of Nursing, University of Indonesia with Number: KET‐186/UN2. F12.D1.2.1/PPM.00.02/2024. Informed consent was obtained from all participants prior to data collection.

## 3 Results

The analysis generated three overarching categories, each comprising several themes (five themes in total). The categories and their associated themes are presented below, with illustrative quotations provided in Table 2.

**TABLE 2:** Sample of data categorization.

Categories	Themes
OHNs duties in mining industry	Direct care
	Non direct care
Barriers and challenges of performance	Internal barriers of OHNs
	Organizational barriers
Competency Development Expectations	Competency‐based Education and Training

### 3.1 Demographic Characteristics of Participants

A total of 20 OHN working in Merdeka Company consented to participate in this study. The average age of the OHNs (*n* = 20) was 39 years (range 23–60) with 13 (65%) participants were males and 11 are registered nurses (55%). About one‐third (38%) had 1–5 years of work experience ranging from less than one year to 37 years (Table 1).

### 3.2 Category One: OHNs Duties in Mining Industry

The analysis generated three overarching categories: OHN duties in mining industry, Barriers and challenges of performance, and competency development expectations. Each category comprised of several themes (Table 2). The results are presented below, with illustrative identified quotations.

#### 3.2.1 Theme 1: Direct Care

Nurse activities in the company aim to carry out occupational health programs that have been prepared by Occupational Health Physician (OHP). Medical check‐ups, emergencies, fit to work, return to work, occupational safety, worker counselling, case managers, and monitoring of high‐risk workers are carried out by OHNs to support the health of mining workers. First, OHNs described that their roles are based on the health problems among miners, such as basic health services and first aid Illustrated below:

We are tasked with supporting occupational health doctors, especially in basic health services, first aid, and occupational health administration. … (Participant 4)

Paramedics are more focused on direct health services… (Participant 7)

In addition to promotive, preventive, curative, and rehabilitative, we also have experience in counseling in collaboration with HR…. (Participant 2)

… Medical check‐up management, our role as a corporate physician assistant, including conducting reviews and providing input, … medical device management, ensuring all equipment is ready. (Participant 3)

Occupational safety, including data collection, report making, and evaluation of the results of activities that have been carried out (Participant 4)

Workers are assessed for readiness before work; an annual routine evaluation is carried out.… (Participant 7)

Second, the participants described the non‐direct care duties also their job desk at mining company. In addition to providing OHN services, OHNs also involved in health administration, company social responsibility (CSR) of health around the company, coordination of Information of Technology (IT) teams and management of medical devices. For example, nurses must carry out CSR activities outside the company environment.

… In addition to medical duties, we are also asked to assist with CSR activities, socialization to the community, or health programs around the company. … (Participant 2)

… I am also involved in a system that must coordinate with the IT team, which is the integration system “Merdeka Sehat”.“ehat”.also invol

Sometimes nurses in the company also act as *case managers*. … (Participant 5)

The paramedic function is sometimes mixed with HR or the general part, especially regarding insurance claims or absenteeism reports due to illness. … (Participant 6)

#### 3.2.2 Theme 2: Indirect Care

In addition to conducting direct nursing activities, OHN also has a responsibility to support the process of improving the health of workers inside and outside the company.

We are also involved in medical device management, ensuring all equipment is ready (Participants 3, 4)

For promotive and preventive programs, such as counseling or health campaigns″ (Participants 2, 5)

### 3.3 Category Two: Barriers and Challenges of Performance

Participants revealed the following barriers and challenges of the work performance which were divided into two themes, namely personal and organizational.

#### 3.3.1 Theme 1: Internal OHNs Barriers

The participants explained that they faced challenges adapting to newly assigned work tasks within his regular workplace duties. Participants try to adjust to the demands of the job by participating in a lot of education and training related to their work demands. In addition, they stated that they experienced limited competencies at work due to a lack of knowledge and skills acquired while undergoing education in college.

Another challenge is also the limitation of competence. … we learn a lot on our own (Participants 2,3,5,6,8)

In addition, three participants also carried out activities that were felt to be too much and overlapped with other tasks in their work as OHNs.

The paramedic function is sometimes mixed with human resources (HR) or the general part, … administrative matters. (Participants 1,7,9)

… sometimes there is still overlap with HR, for example related to sick leave and administrative documents (Participants 2,4,5,6,8)

… Our tasks are many: from administration, medical services, education, to reports to management. Sometimes one person must do it all (Participants 3,10)

The high workload experienced by the participants so that it raises several complaints such as fatigue and difficulty in determining the priority of work that must be completed in a limited time.

Even though we are tired, we feel that we have a great contribution to workers and companies. Sometimes I feel like I'm “all‐round” but that's what makes me tired. (Participants 5,7)

… Sometimes you get confused about which priority to prioritize, because everything feels important. (Participant 2)

… Because we are often required to accomplish many things in a short time… (Participants 4, 5)

### 3.4 Theme 2: Organizational Barriers

The influence of support from organizations is also strongly felt by OHNs in applying occupational health programs. Additionally, participants described experiencing obstacles when adjusting to newly assigned work tasks. This indicates that some OHNs were required to take on responsibilities without adequate preparation, support, or clarification from the organization. Such challenges reflect not only practical difficulties in role adaptation but also raise ethical concerns related to inadequate orientation, unclear job expectations, and the potential for being placed in roles beyond their current competence. According to all participants, successful implementation depends on having supportive organizational policies, such as adequate HR, appropriate budget allocation, and meaningful engagement from organizational leadership. The lack of support is still felt by the participants, especially since there is still a lack of understanding of the business and the organization focuses more on the productivity of the company and curative programs because it considers that the health of workers is not an investment in the company's business. Described in the statement as follows:

…Companies are more concerned with production targets than worker safety or health… (Participants 5,6,8,4,2)

… Many workers still consider health unimportant. They only come to the clinic when they are seriously ill. Even though prevention should be prioritized (Participant 7)

… Making them want to participate in medical check‐ups, counseling, or health programs is not easy. Some are afraid, some are lazy, some feel that it is a waste of time (Participant 4)

A total of 5 (five) participants expressed the attitude of the company's management in determining the priority scale in their organization and still not paying more attention to promotive and preventive efforts.

The support is there, but it focuses more on things that are emergency. For promotive and preventive programs, such as counseling or health campaigns, sometimes they receive less attention because they are considered less urgent (Participant 2)

…Curative programs are always fully supported, but promotive and preventive are often not considered important. … (Participant 4)

We once proposed a routine gymnastics program for workers before shifts. It is good for health and improves fitness… (Participant 7)

Where I once had a monthly health talk program. Initially supported, but over time it was considered not a priority… Health programs are considered a cost, not an investment (Participant 5)

… Management has not seen long‐term benefits, so it is difficult for us to develop the program. (Participant 8)

In addition, two participants stated the lack of resources such as health HR, costs, and medical equipment felt by the participants (see Table 2).

The number of medical personnel is often not proportional to the number of workers. So, we are overwhelmed if there are many cases at once (Participant 2)

¨ resource limitations. Both in terms of the number of medical personnel, equipment, and budget. Sometimes we want to do a good program, but it comes at a cost. (Participant 6)

### 3.5 Category Three: Competency Development Expectations

Nurses in the workplace in this study expressed their hopes for their development as OHNs in mining companies through the development of CE programs both formal and non‐formal. Participants’ expectations regarding CE and competency development are outlined in Table 2. OHNs said that they did not get the curriculum of OHN education in lecture education starting from the Diploma III and Bachelor in Nursing. OHNs hope to have an education with the existence of an occupational health curriculum in nursing education and additional training to support roles in the workplace that have not been felt by participants so that participants feel that they are self‐taught from the workplace. The reason participants felt the need to get a CNE for occupational health was for several reasons.

Formal education today is still more focused on the clinical aspect, while OHNs require broader skills. (Participant 1)

So far, our role has sometimes been underestimated. Even though the contribution is huge. (Participants 2.4)

… Not only ordinary nurses, but nurses must be able to see workers holistically: physically, mentally, socially, and even in their work environment. (Participant 5)

… without a suitable curriculum, it is difficult to prepare a workforce that is truly prepared for the complexities in the field (Participant 8)

this work is both challenging and interesting (Participant 7)

… The OHNs in the future can become a profession that is more recognized, not just a “corporate physician assistant”. … (Participant 2)

In Indonesia, majority of the OHN's experiences in obtaining health competencies is through Industrial Hygiene and Occupational Health training (HIPERKES) organized by a certified Training Institute of the Ministry of Manpower of the Republic of Indonesia which is valid for entire life. However, participants feel that they still need to acquire competencies that are in accordance with the increasingly complex occupational health needs in the company.

… Basic Life Support, First Aid, Health Data Management, to Effective Communication. Because in the field, we are often the first spearhead when there are cases. If you are not prepared, you can be overwhelmed (Participants 7,5,6)

… Counseling and mental health skills. … (Participants 5,6)

…Surrounding Public Health and Disaster Management (Participants 3,6)

… Collaboration skills, cross‐sector cooperation, communication with management, through research, or through scientific publications, administrative skills are also important… (Participant 2,3,4,7,5,6,8)

In addition to communication, administrative skills, IT, data analysis are also important… (Participants 4,8)

… Often we also become a liaison between workers and companies. So, negotiating skill and mediation also important …Often we also be,7)

## 4 Discussion

Based on findings, the experiences and role expectations of OHNs practices in mining industries, barrier and challenges to carrying out OHNs role in mining and expectations for improvement competencies through CE. These findings have three distinct implications for improvement. First, the role and responsibilites as an OHNs in mining sector. Second, issue of limited competence points to the need for structured CE programs, skill‐based training, formalized OHN competency standards, and integration of occupational health content into undergraduate and postgraduate nursing curricula. Strengthening educational preparation would ensure that OHNs enter the workforce with a clear set of foundational skills. Third, the lack of company support reflects an organizational challenge that cannot be resolved solely through individual capacity‐building. Mining companies must allocate adequate resources, provide clear role expectations, offer consistent managerial supervision, and embed OHNs into decision‐making processes related to worker health. Organizational recognition of the OHN role is critical to enabling them to implement comprehensive occupational health programs.

### 4.1 Direct and Indirect OHNs Duties in the Mining

In this study, the form of direct care appears in the routine activities such as conducting morning briefings containing evaluation and service planning, carrying out health promotions, providing first aid in accidents, and conducting post‐illness rehabilitation. In line with some previous research that the direct care role in mining would likely encompass health assessments, mental and physical health support, education on safety and legislation, and coordination of health activities to support both individual miners and organizational health goals (Hatanaka et al. 2020; Pilusa and Mogotlane 2018; Togita and Yano 2024). OHNs contribute to the assessment and monitoring of workers’ health status, including the evaluation of physiological strain through measures like heart rate monitoring during shifts, and the identification of psychological stressors related to work conditions and safety culture (Smith et al. 2022; Varga et al. 2016).

OHNs role and responsibilities is in line with the function of OHNs as stated by (World Health Organization 2001, 2018), which is to provide primary health services in the workplace with a promotive, preventive, curative, and rehabilitative approach. According to Oakley (2008) and Permatasari et al. (2022), one of the functions of OHNs is to manage occupational health services, including setting planning, policy development, funding, and staffing. This includes involvement in operational activities that support teamwork and collaboration, which are essential for delivering healthcare in mining primary healthcare clinics (Nene 2024).

Indirect care responsibilities of OHNs in mining likely encompass organizational assessment, health promotion, coordination of support activities, and professional development to sustain occupational health initiatives, thereby contributing to safer and healthier mining workplaces through systemic and preventive measures (Campbell and Burns 2015; Hatanaka et al. 2020; Kloss 2024; Songkham 2024). From these two themes, it can be seen that the role of OHNs is not only limited to clinical services, but also includes managerial aspects and comprehensive occupational health management. This strengthens the position of OHNs as professionals who have a strategic contribution in maintaining the sustainability of worker productivity while supporting the company's goals in terms of occupational safety and health.

### 4.2 Barriers and Challenges to Carrying out OHNs Role at Mining Industry

The results of this study also reveal the barriers and challenges faced by OHNs to carrying out their roles in the company and divided into two main factors, namely the limited competence and the lack of company support. Firstly, competency limitations arise because most nurses do not have a specific formal educational background in the field of OHNs. As a result, mastery of specific concepts, principles, and skills such as occupational health risk management, worker health surveillance, and work environment evaluation is still perceived to be less than optimal. The limitation of competency in OHNs is closely tied to regulatory deficiencies, lack of clear professional standards, and challenges in integrating research into practice, arise from structural, regulatory, epistemological, and practical challenges that affect professional recognition, leadership, evidence‐based practice, and adaptability to workplace contexts (González‐Caballero 2023; 2024; Onyeador and Umberger 2023; Songkham 2024; Takeguchi 2022). This condition makes nurses learn more through direct experience (learning by doing) than through formal education channels. This is in line with the findings of Salazar (2006) that competencies in OHN often develop progressively through direct involvement, but are not supported by an adequate formal curriculum. Therefore, ensuring OHNs have adequate education, training, and supportive work environments is essential to prevent negative consequences related to competency limitations  (Almarwani and Alzahrani 2023; Arnetz et al. 2025; Onyeador and Umberger 2023).

In contrast, lack of company support represents an organizational barrier that cannot be resolved solely through individual skill development. Company policies did not adequately support the role of nurses in the workplace. Participants reported that management prioritized business operations over occupational health concerns, often failing to respond to recommendations from nurses and occupational safety and health teams. They also noted that workplace facilities were insufficient to support nurses in performing their responsibilities effectively. This support includes the provision of occupational health infrastructure, access to training programs, and recognition of the role of nurses in the company's health and safety management system. The lack of support causes OHNs to often feel fatigue and they are working alone without an adequate support system, both structurally and institutionally. This condition is in line with the statement of WHO (2017) that an unsupportive work environment will limit the role of occupational health workers in achieving optimal service standards. Negative organizational culture and politics correlate with higher levels of work‐related stress, while positive organizational factors are associated with reduced stress and greater workplace satisfaction (Nantsupawat et al. 2017). Poor work environments have also been shown to increase nurses’ intention to leave their positions, further affecting workforce stability (Kiptulon et al. 2024). These findings underscore the importance of fostering organizational support to enhance job satisfaction among OHNs and other nursing professionals (Ahmad et al. 2022; Larsman et al. 2024; Pahlevan Sharif et al. 2018; Poghosyan et al. 2020). Participants described inadequate staffing, insufficient resources, minimal managerial involvement, and limited recognition of the OHN role. These challenges require organizational action, such as increasing budget allocation for occupational health programs, clarifying the role of OHNs within company policies, ensuring adequate staffing levels, providing access to training, strengthening collaboration between management and OHNs, and integrating OHNs into decision‐making processes related to workers health.

### 4.3 Continuing Nurse Education Expectations

In this study, OHNs are expressed expectations related to their self‐development as well as their profession through CE. These findings show a gap between the need for competency development of OHNs in the field and the availability of regulatory and institutional support. CE is viewed as a means to support long‐term careers and work‐life balance for OHNs, contributing to workforce stability and growth. These findings are in line with the statement of Schwarz and Lindfors (2015) who stated that although the involvement of nurses in nursing tasks with moderate intensity can be beneficial for maintaining health status, the intensity, duration, and frequency of such involvement are not sufficient to produce a more optimal training experience for nurses. The reason need for CNE regarding Salazar (2006) explained that the core curriculum in health care, occupational safety, and the environment contains concepts and principles that are the basis of knowledge for occupational health and environmental nursing practices.

OHNs perceive CE as valuable for professional advancement and workforce retention, but their engagement is affected by awareness and confidence in accessing educational funding and opportunities (Weinsier and Garcia‐Cameron 2025). Addressing these challenges can enhance OHNs’ ability to pursue CE, thereby supporting their professional growth and contribution to workplace and community health (De Castro et al. 2015; You et al. 2025). Similar to the findings of Prasetyono et al. (2024), OHNs in Indonesia are still require training to improvement competencies such as research or documentation, clinical task, promoting resilience and communication collaboration. Another research identifies seven key competencies necessary for effective OHN practice: self‐growth, perpetuation of OHN essence, strategic planning and duty fulfilment, coordination, client growth support, team empowerment, and creativity (American Association of OHNs (AAOHN) (Kono et al. 2017). The AAOHN itself has historically advocated for quality undergraduate and graduate education, certification, and the development of practice standards and competencies, underscoring the ongoing need for structured professional development and research dissemination in the field (Wachs 2017). OHNs competencies in the new century similar finding with this research, it is include clinical care, mental health promotion, organizational problem‐solving, continuous professional development, leadership, and political engagement, reflecting a multifaceted role that integrates individual and systemic health perspectives (De Jager et al. 2016; González‐Caballero 2025; Lalloo et al. 2016; Takeguchi 2022; Togita and Yano 2024). OHNs in modern workplaces need competencies in assessment‐based planning, collaboration, organizational system development, political engagement, clinical judgment, communication, and self‐care to effectively meet the demands of their roles (Kanamori et al. 2025).

Together, these findings suggest that both individual and organizational level interventions are necessary to improve worker health outcomes. Recommendations include developing national OHN competency standards, expanding OHN curricula, strengthening employer policies that support OHN practice, improving staffing ratios, and establishing structured CE requirements. Therefore, increasing the capacity of OHNs through CNE formal or non‐formal education, as well as strengthening institutional support from mining companies, is a crucial step to ensure the quality of occupational health services in the future.

### 4.4 Limitations

Qualitative data is limited to information provided by participants in FGDs. Using a focus group may have restricted the range of perspectives obtained, and group dynamics may have influenced how openly participants shared their experiences. Some individuals may have been reluctant to speak candidly in front of colleagues or may have feared workplace consequences for expressing critical views. Additionally, dominant participants may have shaped the discussion, while quieter participants contributed less. As a result of the fact that this study was conducted on mining companies in Indonesia, transferability to a population of OHNs with different working environments cannot be generalized from this study.

## 5 Conclusions

The study examined nurses' perceptions and expectations to enhance competencies through CNE education and training for supporting nursing practices in mining industries. Based on the results of the study, OHNs still experience limitations in their work performance in practicing their duties as occupational health service providers in the mining industry. Although the majority of OHNs have participated in certified occupational health training (HIPERKES) activities organized by the Ministry of Manpower of the Republic of Indonesia, they still consider that additional training is needed to minimize the competency gap at work. The provision of an OHN curriculum while still in college is one of the strategies to prepare nursing graduates who are ready to work in the mining company environment. In addition, through continuous training, OHNs will also be able to help to minimize the limitations of competencies that have been experienced in the workplace. Competencies in assessment‐based planning, collaboration, clinical judgment, communication, negotiation, counseling, health data analysis and management as well as the implementation of basic practices of OHN need to be trained so that the role of OHNs can be felt to support the productivity of companies.

### 5.1 Implications

The findings of this study highlight importantly of the experiences CNE among OHNs in mining industry. Beyond implications for nursing education and individual OHN development, these findings also point to the need for action at the organizational and policy levels. Nursing education in universities should have an OHN curricula to prepare students on various occupational health practices. Employers play a critical role in supporting effective OHN practice by allocating adequate resources, ensuring sufficient staffing, providing structured orientation and ongoing training, and integrating OHNs into decision‐making processes related to worker health and safety. At the policy level, regulators such as Ministry of Health could strengthen national standards for OHN competencies, clarify minimum resource requirements for workplace health units in mining, and enhance monitoring of occupational health program implementation. These findings also highlight the need for further research to examine OHNs practice across diverse mining contexts, evaluate the effectiveness of competency building interventions, and explore the impact of organizational support on worker health and public health outcomes.

## Authors’ Contributions

All authors contributed to the study conception and design. Material preparation, data collection, and analysis were performed by JP and MM. HP contributed to the methodological design and supervised data collection. AS supported data validation and interpretation of findings. SM dan TS assisted in conceptual framework development and manuscript revision. MM, HP, AS, provided overall supervision and critical review of the manuscript. The first draft of the manuscript was written by JP, and all authors commented on previous versions of the manuscript. All authors read and approved the final manuscript.

## Funding

This study was supported by the University of Eastern Finland (Itä‐Suomen Yliopisto) and Indonesian Education Scholarship, Center for Higher Education Funding and Assessment, and Indonesian Endowment Fund for Education. The funding body had no role in the study design, data collection, analysis, interpretation, or manuscript preparation.

## Ethics Statement

This study received ethical approval from the Ethics Commission of the Faculty of Nursing, University of Indonesia with Number: KET‐186/UN2. F12. D1.2.1/PPM.00.02/2024. Participants were required to have adequate information about the research, comprehend that information and have the choice to voluntarily consent or decline participation (Polit & Beck 2017:521).

## Conflicts of Interest

The authors declare no conflicts of interest.

## Data Availability

The qualitative data generated and analysed during the current study are not publicly available due to confidentiality agreements with participants but are available from the corresponding author on reasonable request.
